# Combining treatment effects from mixed populations in meta-analysis: a review of methods

**DOI:** 10.1186/s12874-025-02507-3

**Published:** 2025-04-02

**Authors:** Lorna Wheaton, Sandro Gsteiger, Stephanie Hubbard, Sylwia Bujkiewicz

**Affiliations:** 1 Biostatistics Research Group, Department of Population Health Sciences, University of Leicester, University Road, Leicester, LE1 7RH UK; 2 Global Access, F Hoffman-La Roche AG, Basel, Switzerland

**Keywords:** Meta-analysis, Biomarker, Subgroup

## Abstract

**Background:**

Meta-analysis is a useful method for combining evidence from multiple studies to detect treatment effects that could perhaps not be identified in a single study. While traditionally meta-analysis has assumed that populations of included studies are comparable, over recent years the development of precision medicine has led to identification of predictive genetic biomarkers which has resulted in trials conducted in mixed biomarker populations. For example, early trials may be conducted in patients with any biomarker status with no subgroup analysis, later trials may be conducted in patients with any biomarker status and subgroup analysis, and most recent trials may be conducted in biomarker-positive patients only. This poses a problem for traditional meta-analysis methods which rely on the assumption of somewhat comparable populations across studies. In this review, we provide a background to meta-analysis methods allowing for synthesis of data with mixed biomarker populations across trials.

**Methods:**

For the methodological review, PubMed was searched to identify methodological papers on evidence synthesis for mixed populations. Several identified methods were applied to an illustrative example in metastatic colorectal cancer.

**Results:**

We identified eight methods for evidence synthesis of mixed populations where three methods are applicable to pairwise meta-analysis using aggregate data (AD), three methods are applicable to network meta-analysis using AD, and two methods are applicable to network meta-analysis using AD and individual participant data (IPD). The identified methods are described, including a discussion of the benefits and limitations of each method.

**Conclusions:**

Methods for synthesis of data from mixed populations are split into methods which use (a) AD, (b) IPD, and (c) both AD and IPD. While methods which utilise IPD achieve superior statistical qualities, this is at the expense of ease of access to the data. Furthermore, it is important to consider the context of the decision problem in order to select the most appropriate modelling framework.

**Supplementary Information:**

The online version contains supplementary material available at 10.1186/s12874-025-02507-3.

## Background

Evidence synthesis is a branch of statistical methods which allows researchers to combine multiple sources of evidence in a single analysis. This allows for a larger body of evidence, compared to individual studies, to be used and thus can lead to more precise treatment effect estimates. One common method of evidence synthesis is meta-analysis of studies addressing the same research question, in the same population and using the same statistical design.

Meta-analysis assumes that the treatment effects in each included study are somewhat comparable to each other, with fixed-effect models assuming underlying treatment effects are identical for each study and random-effects models assuming treatment effects estimated in each study come from a common normal distribution. However, over recent years there has been increasing interest in the field of precision medicine which identifies subgroups of a population, for example by genetic biomarkers, to which targeted therapies can be delivered successfully which in turn can reduce failure rates in randomised controlled trials (RCTs). This can reduce the time and cost of the drug development process. Such biomarkers are known as predictive biomarkers as treatment effect depends on the biomarker status of the patient. Predictive biomarkers, such as genetic biomarkers, are sometimes identified in post-hoc exploratory analyses of trials.

When predictive biomarkers, such as genetic biomarkers, are used to identify subsets of a population which can benefit from treatment targeted on those biomarkers, they are investigated in clinical trials. Such trials are often of mixed designs resulting in mixed patient populations within treatment arms across trials [1]. For example, Cetuximab and Panitumumab were originally approved (in 2004 and 2006 respectively) to target the epidermal growth factor receptor (EGFR) which may be overexpressed in patients with metastatic colorectal cancer (mCRC) [2]. However in 2009, retrospective analysis of trials stratified by KRAS mutation status found that patients with KRAS mutations (MT) did not achieve improved survival when treated with EGFR-targeted therapies compared to being treated with chemotherapy [3–5]. This resulted in Cetuximab and Panitumumab no longer being recommended to mCRC patients with KRAS mutations and subsequent trials often investigated effectiveness of these therapies in KRAS wild-type (WT) patients alone [2]. Therefore, the evidence base developed over the years on the effectiveness of Cetuximab and Panitumumab has consisted of trials with mixed populations with some trials investigating KRAS WT and MT patients with no subgroup analysis, some trials investigating KRAS WT and MT patients with subgroup analysis and some trials only investigating KRAS WT patients [6–8]. Such development of Cetuximab and Panitumumab has generated difficulties for synthesis of data in the population of interest using standard meta-analytic methods. The aim of this paper is to review and describe novel meta-analytic methods for synthesis of data on treatment effectiveness in mixed populations.

In this paper, we first give a brief background of standard meta-analysis methods in the Background methods section. These core methods are then built upon by the identified methods later in the paper. In Identification of methodological work, we describe the process of the identification of previously published methodological work. In Identified methods, we describe the identified methods for evidence synthesis of mixed populations which build on the base methods described in the Background methods section. In Comparison of methods, we apply a selection of the methods to an illustrative example in mCRC. Finally, we provide a discussion of the identified methods and present our conclusions.

## Background methods

The methods identified in our review (and described in Identification of methodological work) build on a range of standard meta-analytic methods. This section provides an overview of these standard meta-analytic methods introducing common terminology and notation. The background methods are described in a Bayesian framework since the majority of the identified methods utilise the Bayesian approach. However, these methods can equally be applied in a frequentist framework. This section is designed to outline key concepts without extensive technical details, allowing readers unfamiliar with these methods to gain an understanding of the broader context. For those requiring a more thorough introduction to meta-analysis, please refer to Chapter 10 of the Cochrane Handbook for Systematic Reviews of Interventions [9] and Individual Participant Data Meta-Analysis: A Handbook for Healthcare Research [10] for a comprehensive introduction to aggregate and individual-level data meta-analytic methods. These introductions can be complemented by the texts referenced throughout this section.

### Aggregate data meta-analysis (ADMA)

#### Fixed-effect and random-effects meta-analysis

Traditionally, pairwise meta-analysis has been applied to aggregate data (AD) extracted from published trial reports, using trial-level data to compare two treatments and estimate the pooled treatment effect. Pairwise AD meta-analysis (ADMA) can assume either fixed-effect or random-effects. Assuming we have normally distributed treatment effects in a fixed-effect model (see Appendix 1 for binomially distributed outcomes) observed treatment effects ($\hat{\delta _{i}}$) across studies only differ due to random error, assuming a common underlying “true” effect ($\delta$):

1 $$\begin{aligned} \hat{\delta }_{i} \sim N(\delta , \sigma _{i}^2) \end{aligned}$$

where $\sigma _{i}^2$ is the within-study variance in study *i* which is assumed known.

However, an assumption of a fixed-effect is often unrealistic as studies can differ in a variety of ways; such as being conducted in different locations, or using heterogeneous populations which can lead to varying underlying “true" treatment effects between studies [11, 12]. Instead, a random-effects model assumes that the “true" treatment effect in each study *i* ($\delta _{i}$) can differ across studies. The “true" treatment effects are then assumed to come from a common distribution with a mean, *d*, and between-study variance $\tau ^2$:

2 $$\begin{aligned} \hat{\delta }_{i}&\sim N(\delta _{i}, \sigma _{i}^2) \end{aligned}$$

3 $$\begin{aligned} \delta _{i}&\sim N(d, \tau ^2) \end{aligned}$$

When fixed-effect or random-effects meta-analysis is conducted in a Bayesian framework, prior distributions are required on *d* and $\tau$ [13].

#### Meta-regression

Random-effects meta-analysis has traditionally been used when significant heterogeneity is observed between studies. However it only accounts for, rather than explains, these differences. Meta-regression was developed to explicitly model heterogeneity across subgroups or a range of covariate values. Meta-regression is a single integrated analysis with a shared between-trial heterogeneity parameter and a treatment-covariate interaction term showing the effect of a covariate on the treatment effect:

4 $$\begin{aligned} \hat{\delta }_{i}&\sim N(\delta _{i}, \sigma _{i}^2) \end{aligned}$$

5 $$\begin{aligned} \delta _{i}&\sim N(\alpha + \beta z_{i}, \tau ^2) \end{aligned}$$

where $z_{i}$ is the trial-level covariate for study *i* and $\beta$ gives the interaction between the covariate and treatment effect. This covariate can represent a subgroup, continuous covariate or baseline risk.

However, there are several disadvantages of using meta-regression. First, meta-regression can be subject to ecological, or aggregation, bias whereby the relationship observed between the covariate and outcome differs between the individual-level and the aggregate-level [14]. Second, the results of a meta-regression are easier to interpret when for a given covariate there is a wide range of values across studies [15]. Third, meta-analyses often contain few studies meaning that it is difficult to obtain robust conclusions from meta-regression [16].

### Individual participant data meta-analysis (IPDMA)

The methods described thus far utilise AD only. However, it is generally accepted that the gold standard for meta-analysis is to conduct an individual participant data meta-analysis (IPDMA). IPD has the potential to improve the quality and scope of data included in the meta-analysis, allowing for adjustment of relevant prognostic factors and standardisation of analysis at the trial-level [17, 18]. There are two main approaches for conducting IPDMA; the one-stage and the two-stage methods.

#### One-stage and two-stage approaches

In the two-stage model, the first stage is to analyse the IPD from each trial separately in order to obtain a treatment effect estimate ($\hat{\delta _{i}}$) and within-study variance ($\sigma _{i}^{2}$) of this treatment effect estimate. The model used to analyse the data in the first stage of the two-stage approach will vary depending on the outcome. For example a linear regression could be used to model continuous outcomes and logistic regression to model binary outcomes. In the second stage, treatment effect estimates are combined in the same way as for a conventional ADMA (Eqs. (1) & (2)). The ability to access IPD to conduct analysis for each trial allows for (a) standardisation of inclusion and exclusion criteria across studies, (b) standardisation of outcome definitions and (c) use of uniform statistical methods for analysis at the trial-level [17, 19].

Alternatively, the one-stage model analyses IPD from all studies simultaneously using a hierarchical regression model:

6 $$\begin{aligned} y_{ij} \sim N(\alpha _{i} + \delta _{i}x_{ij}, \sigma _{i}^2)\end{aligned}$$

7 $$\begin{aligned} \delta _{i} \sim N(d, \tau ^2) \end{aligned}$$

where $y_{ij}$ and $x_{ij}$ are the observed outcome and treatment arm respectively for participant *j* in study *i*, $\alpha _{i}$ is the expected outcome in study *i* when all covariate values are zero (here the only covariate is treatment arm) and $\delta _{i}$ is the treatment effect in study *i*. As a random-effect on the treatment effect is being used, $\delta _{i}$ is assumed to come from a normal distribution with a mean *d*, representing the summary treatment effect, and a variance $\tau ^2$, representing the between-study variance. When conducting a one-stage IPDMA using a Bayesian approach, prior distributions on *d* and $\tau$ are required.

A benefit of the one-stage approach is that it provides a flexible approach for synthesis of data, allowing common, random or stratified parameters at the discretion of the researcher. Furthermore, the one-stage approach models the exact likelihood at the individual level. This is in contrast to the second stage of the two-stage approach which assumes that treatment effect estimates are normally distributed with known variances which can be inappropriate for small studies or studies with few events [20]. However, it is important to note that in most cases, provided the same model specification is used, the same results will be obtained regardless of whether a one-stage or a two-stage model is used [21].

#### IPDMA investigating treatment-covariate interactions

Meta-regression to model heterogeneity between subgroups, as described in the Meta-regression section, assumes that across-trial interactions will accurately reflect within-trial interaction. However, meta-regression can suffer from aggregation bias and has low power to detect genuine treatment-covariate interactions. In contrast, IPDMA is particularly useful when investigating treatment-covariate interactions as it can investigate the treatment-covariate interaction at the individual-level [22–24].

A two-stage IPDMA can estimate treatment-covariate interactions by first modelling the within-study treatment-covariate interaction, followed by pooling these effects across studies using either a fixed-effect or random-effects model. A one-stage approach can also be used to estimate treatment-covariate interactions. However, it should be noted that the one-stage approach is more complicated than the two-stage approach when investigating interaction terms. Simply including an interaction term in the one-stage approach can risk amalgamating across-trial and within-trial information. Not fully separating out the across-trial and within-trial effects can lead to bias and therefore potentially misleading results about whether there is a treatment-covariate interaction at the participant-level. To avoid this problem two methods are recommended by Riley et al. [10]; the first is to centre the covariate, $z_{ij}$, around its trial-specific mean, $\bar{z}_{i}$ and add an additional term which allows the covariate means to explain between-trial heterogeneity in the treatment effect and the second is to stratify by trial all other parameters outside the interaction term.

### Extensions

#### Network meta-analysis (NMA)

All methods described in this section, whether using AD or IPD, have compared two treatments in pairwise meta-analysis. However, decision-makers often want to compare more than two treatments in order to answer questions such as “which treatment is most effective overall". Network meta-analysis (NMA) is an extension of pairwise meta-analysis which allows the synthesis of data on more than two interventions [25]. A brief description of NMA for continuous and binomial data is available in Appendix 2.

#### Combining AD and IPD

While IPDMA is considered the gold standard, in many cases researchers are unable to obtain IPD for all studies [23]. Inability to obtain IPD for all eligible studies could be a result of an inability to contact study authors, unwillingness to collaborate by study authors or loss of IPD. A review of 175 IPDMA articles found that 29% of articles only obtained IPD for less than 80% of the studies [23]. The inclusion of fewer studies in the IPDMA will reduce the power of the analysis and furthermore, if the inability to access IPD is related to the study results, this can lead to bias in the results of the IPDMA [17]. To mitigate potential issues of low power and bias, methods have been developed to combine AD and IPD in a single meta-analysis. A brief summary of these methods are available in Appendix 3.

## Identification of methodological work

We systematically searched through PubMed to identify methodological papers on evidence synthesis for mixed populations using the search filters supplied Appendix 4. We restricted the search to main applied statistical journals with a focus on applications in life sciences and medicine. These included the following: Biometrics, Biometrical Journal, Biostatistics, Journal of the Royal Statistical Society series A and C, Research Synthesis Methods, Statistics in Medicine and Statistical Methods in Medical Research. We also included main methodological journals, which publish applied work, which are the following: Annals of Applied Statistics, Journal of the American Statistical Association, Journal of the Royal Statistical Society series B and Statistical Science. Articles were considered eligible for inclusion if they described statistical methods for analysing AD or IPD from mixed populations in a single meta-analysis or network meta-analysis (NMA).

## Identified methods

**Fig. 1:**
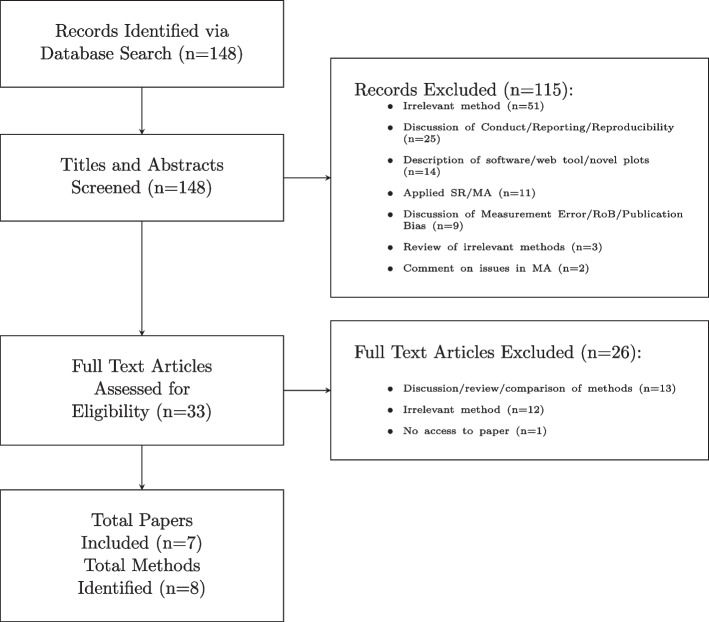
Flowchart of the literature review and paper and methodology selection process

We identified eight methods for evidence synthesis of mixed populations (see Fig. 1). Three methods are applicable to pairwise meta-analysis using AD, three methods are applicable to network meta-analysis using AD and two methods are applicable to network meta-analysis using AD and IPD. We discuss each of the identified methods in turn, starting with methods for AD pairwise meta-analysis, extending to methods for AD NMA and finally looking at methods for AD and IPD NMA.

### Wheaton et al. method

Wheaton et al. [26] describe an ADMA method for utilising data from studies investigating biomarker-positive, biomarker-negative and biomarker-mixed populations in order to improve precision of estimation of treatment effects on the biomarker subgroup of interest (in the example this was biomarker-positive patients). Code to implement this model using the WinBUGS software is available in the supplementary material of the manuscript describing the method. In this model, treatment effects obtained from analysis of biomarker-positive patients ($\hat{\delta }_{+i}$) are analysed using a standard random-effects meta-analysis model:

8 $$\begin{aligned} \hat{\delta }_{+i} \sim N(\delta _{+i}, \sigma _{+i}^2) \end{aligned}$$

9 $$\begin{aligned} \delta _{+i} \sim N(\gamma _{+}, \tau _{+}^2) \end{aligned}$$

where vague prior distributions are placed on $\gamma _{+}$ and $\tau _{+}$.

To incorporate treatment effects from analysis of biomarker-negative patients ($\hat{\delta }_{-i}$), it is assumed there is a systematic difference in treatment effects between biomarker-positive and biomarker-negative patients:

10 $$\begin{aligned} \hat{\delta }_{-i} \sim N(\delta _{-i}, \sigma _{-i}^2) \end{aligned}$$

11 $$\begin{aligned} \delta _{-i} = \delta _{+i} + \beta _{i} \end{aligned}$$

where $\beta _{i}$ is the systematic difference which is allowed to vary between studies but is assumed to come from a common normal distribution with a mean, $\mu _{\beta }$, and variance, $\tau _{\beta }^2$:

12 $$\begin{aligned} \beta _{i} \sim N(\mu _{\beta }, \tau _{\beta }^2) \end{aligned}$$

To incorporate treatment effects from analysis of biomarker-mixed patients ($\hat{\delta }_{mix,i}$), it is assumed that the treatment effects in biomarker-mixed patients are equal to the treatment effects in biomarker-positive patients plus the systematic difference, $\beta _{i}$, multiplied by the proportion of biomarker-negative patients in the biomarker-mixed study, $p_{i}$:

13 $$\begin{aligned} \hat{\delta }_{mix,i} \sim N(\delta _{mix,i}, \sigma _{mix,i}^2) \end{aligned}$$

14 $$\begin{aligned} \delta _{mix,i} = \delta _{+i} + p_{i}\beta {i} \end{aligned}$$

The main advantage of this model is that it allows the treatment effects on biomarker-positive patients concealed within studies investigating biomarker-mixed populations, which do not report subgroup analysis, to be interpolated based on the systematic difference in treatment effects between biomarker-positive and biomarker-negative subgroups. A simulation study indicated that this model is able to improve precision of estimation of the pooled treatment effect in the biomarker-positive subgroup. Other benefits of this model include that it is Bayesian allowing for informative prior distributions to be included and can be easily adapted to include other types of data beyond those which are normally distributed. However, a limitation of this model is that it does not presently extend to cases where there is more than one relevant biomarker of interest. A further limitation of this model is that when the treatment effect is a non-collapsible measure (such as log hazard ratios or log odds ratio) as the mean and variance of the systematic difference increases, bias is introduced into the estimate of the pooled treatment effect, indicating that this model may not work well when there are large and variable differences in treatment effects between subgroups. However, the simulation study also suggested that this limitation was ameliorated when treatment effects from mixed populations were adjusted for biomarker status.

### Linear mixed models

Sørensen et al. [27] describe a *Linear Mixed Model* (*LMM*) for estimating treatment effect modification in ADMA while allowing for missing subgroup information. Code to implement the “basic" model described in Eq. (15) using either R or SAS is available in the supplementary material of the manuscript describing the method. The “basic" *LMM* for estimating the within-study interaction effect when information is available from all subgroups is as follows:

15 $$\begin{aligned} \hat{\delta }_{ki} = \mu + \alpha _{k}x_{ki} + B_{i} + G_{ki} + \epsilon _{ki}, \end{aligned}$$

where $\mu$ is the treatment effect in the reference subgroup, $\alpha _{k}$ is the interaction effect compared with the reference subgroup and $x_{ki}$ is the indicator of subgroup *k*. $B_{i}$ is a study-level random effect, $G_{ki}$ is a subgroup-level random effect and $\epsilon _{ki}$ is the error term:

16 $$\begin{aligned} B_{i}&\sim N(0, \tau ^2) \end{aligned}$$

17 $$\begin{aligned} G_{ki}&\sim N(0, \tau _{k}^2) \end{aligned}$$

18 $$\begin{aligned} \epsilon _{ki}&\sim N(0, \sigma _{ki}^2) \end{aligned}$$

The authors describe how *LMM* can be extended to account for potential effects of ecological bias by including an additional term, $\beta p_{i}$ where $p_{i}$ is the proportion of participants in a particular subgroup meaning that $\beta$ is the change in treatment effect as a function of the study-specific subgroup fraction $p_{i}$. If there is any dependence between treatment effect and the ecological level of the subgroup, the inclusion of the $\beta$ term removes this dependence from the interaction term $\alpha _{k}$.

Studies which do not report all subgroups can easily be included in the *LMM* model. The authors also further extend the model to allow for a systematic difference in treatment effects between studies reporting treatment effects in all subgroups and studies reporting treatment effects in only a subset of subgroups.

Finally, studies which do not provide any subgroup specific treatment effects can be included in the *LMM*. Consider there are studies $I^{SG}$ and $I^{P}$ where $I^{SG}$ is the set of studies reporting subgroup-specific treatment effects and $I^{P}$ is the set of studies reporting only pooled treatment effects. Pooled treatment effects can be included via the following model:

19 $$\begin{aligned} \hat{\delta }_{ki}&= \mu + \alpha _{k}x_{ki} + B_{i} + G_{ki} + \epsilon _{ki} \qquad \text {for} \ i \in I^{SG} \end{aligned}$$

20 $$\begin{aligned} \hat{\delta }_{i}&= \mu + \sum _{k} \alpha _{k}p_{ki} + B_{i} + \sum _{k} G_{ki}p_{ki} + \epsilon _{i}^* \qquad \text {for} \ i \in I^{P} \end{aligned}$$

where $\epsilon _{i}^* = \sum _{k} \epsilon _{ki}p_{ki}, i \in I^{SG}$ refers to studies which are in $I^{SG}$ and $p_{ki}$ is the proportion of participants in subgroup *k* out of the entire population.

As for the method proposed by Wheaton et al., the key advantages of this model are that the model only requires AD to be implemented, the ability to include studies with partially reported subgroups and the ability to include studies which only provide the pooled treatment effect. However, the *LMM* framework also allows for extension beyond two subgroups.

### Extended within-trial framework

Godolphin et al. [28] describe an ADMA or IPDMA method for extending the within-trial framework described in the IPDMA investigating treatment-covariate interactions section and have written the Stata package **metafloat** [29] to allow for implementation of their model in Stata. The within-trial framework for estimating treatment-covariate interactions has recently become the gold standard for assessing how treatment effects vary across participant subgroups. It has been highlighted that techniques such as meta-regression are subject to aggregation bias and conducting separate subgroup analyses and subsequently comparing these pooled treatment effects combines within-trial and across-trial treatment effects which could mask the true within-trial treatment effect [14, 24].

To obtain an estimate of the within-trial interaction alone, it is recommended that the interaction effect between treatment and the subgroup of interest - here biomarker status - is calculated for each trial in the meta-analysis and these interaction terms are meta-analysed. However, there are several limitations of this approach. First, where a treatment-covariate interaction is identified there is no pooled estimate of the treatment effect in each of the subgroups of interest. This is required by policymakers and clinicians for decision-making. Second, the within-trial framework is unable to incorporate studies where the interaction cannot be estimated. This could potentially exclude many useful studies such as older studies where the subgroup was not measured and newer studies which were only conducted in a single subgroup. Third, this model does not extend to scenarios where there are more than two subgroups.

The *Extended Within-Trial Framework* described by Godolphin et al. [28] provides an extension to the within-trial framework by providing methods to estimate within-trial interactions across two or more subgroups and estimate subgroup specific treatment effects which are compatible with these within-trial interactions. The extended model allows for the incorporation of trials conducted in a single subgroup as well as those conducted in mixed populations to make maximum use of the available data.

Suppose there are *n* studies split into *k* subgroups. To simplify the model, assume that there are only two subgroups; biomarker-positive and biomarker-negative patients.

Assuming the full set of subgroup-specific treatment effects are observed for every trial, the standard multivariate meta-analysis model is:

21 $$\begin{aligned} \hat{\beta }_{i} \sim MVN (\beta , S_{i}+\Sigma _{\beta }) \end{aligned}$$

where $\hat{\beta }_{i}$ is the vector of subgroup specific treatment effects in trial *i*, which is assumed to come from a multivariate normal distribution where $\beta$ which is a vector of subgroup-specific treatment effects, $S_{i}$ is the within-study covariance matrix and $\Sigma _{\beta }$ is the between-trial heterogeneity covariance matrix associated with $\beta$.

The interaction between treatment and covariate from trial *i* can then be represented by the $k-1$ treatment effect contrasts $\hat{\gamma }_{i}$ with covariance matrix $V_{i}$:

22 $$\begin{aligned} \hat{\gamma }_{i} \sim MVN (\gamma , V_{i} + \Sigma _{\gamma }) \end{aligned}$$

These effect vectors are referred to as within-trial interaction vectors and are derived from $\hat{\beta }_{i}$ and $S_{i}$ via a simple linear combination which is represented by the contrast matrix ${\textbf {M}}$:

23a $$\begin{aligned}&\hat{\gamma }_{i}={\textbf {M}}\hat{\beta }_{i} \end{aligned}$$

23b $$\begin{aligned}&V_{i}=Var(\hat{\gamma }_{i})={\textbf {M}}S_{i} {\textbf {M}}^T \end{aligned}$$

Typically covariances between the subgroup-specific treatment effects at the trial level will not be reported and can be assumed to be negligible.

In the within-trial framework the contrasts of the subgroup-specific estimates, $\hat{\beta }$, should be equal to the pooled within-trial interactions, $\hat{\gamma }$. Let $\hat{\theta }$ be the subgroup-specific treatment effect estimate in the reference subgroup and thus $\hat{\beta }$ is parameterised as $\hat{\theta }$ plus the $k-1$ estimated contrasts $\hat{\gamma }$ with respect to that reference:

24a $$\begin{aligned} & \hat{\beta }=1_{(k)}\hat{\theta }+\begin{bmatrix}0\\ \hat{\gamma }\end{bmatrix}=1_{(k)}\hat{\theta }+{\textbf {Z}}\hat{\gamma } \end{aligned}$$

24b $$\begin{aligned} & {\textbf {M}}\hat{\beta }=\hat{\gamma } \end{aligned}$$

24c $$\begin{aligned} & \hat{Var}(\hat{\gamma })={\textbf {M}}\hat{Var}(\hat{\beta }){\textbf {M}}^{T}, \Sigma _{\gamma }={\textbf {M}}\Sigma _{\beta } {\textbf {M}}^{T} \end{aligned}$$

Therefore $\hat{\beta }$ is described as the floating subgroup-specific effect estimates.

This model can be specified as a fully common effect model where the interaction terms and subgroup-specific treatment effects are common across studies by setting $\Sigma _{\gamma }=0$ and $\Sigma _{\beta }=0$. However, it is also possible to employ exchangeable random-effects which assumes that heterogeneity variances and covariances do not depend on which subgroups are being compared or unstructured random-effects which allows a different heterogeneity variance to be estimated within each subgroup.

If any estimates $\hat{\beta }_{ji}$ are unobserved for trial *i*, then $\hat{\beta }_{i}$ and $\hat{\gamma }_{i}$ contain estimates just for the observed subgroups. When doing this, the unobserved estimates can be considered to be very imprecisely estimated, for example by assigning them to a value of zero for the effect size and a variance much exceeding the largest observed variance.

Advantages of this model include first, the ability to simultaneously estimate treatment-covariate interactions and treatment effects in subgroups without aggregation bias, second the ability to include studies conducted in a single subgroup and third the ability to apply the model to scenarios where there are more than two subgroups. However, this model also has limitations. First, the authors suggest that the method can be applied using AD alone. However, they acknowledge that in practice, subgroup-specific treatment effects and interactions are unlikely to be presented in published reports for all relevant studies in a meta-analysis. Therefore, in practice this method would likely require IPD. Second, to include studies including only a single subgroup it is necessary to assume transitivity across subgroups. If this assumption is not appropriate it could potentially bias the estimates of the floating subgroup-specific treatment effects.

### Enriching through weighting

The *Enriching Through Weighting* approach was initially presented by Efthimiou et al. [30] for the integration of randomised and non-randomised evidence in network meta-analysis and was translated to a setting looking at integration of studies investigating different subgroups by Proctor et al. [31]. Proctor et al. do not provide code to implement this model. In this setting there are studies which are investigating the biomarker subgroup of interest, biomarker-positive patients, and studies where patients are biomarker-mixed. The model is based on the standard NMA model for binomial data [32], which can be defined as:

25 $$\begin{aligned} r_{it} \sim Bin(p_{it}, n_{it}) & \\ logit(p_{it})= \left\{ \begin{array}{lr} \mu _{ib} & \text {for}\ t=b\\ \mu _{ib} + \delta _{ibt} & \text {for} t\neq b\\ \end{array}\right. & \\ \delta _{ibt} \sim N(d_{bt}, \tau ^2) & \end{aligned}$$

where the indices *i* and *t* indicate study and treatment arm respectively. $r_{it}$ denotes the number of observed events and $n_{it}$ denotes the number of individuals per study arm. $\mu _{ib}$ are the log odds of an event for the baseline treatment and $\delta _{ibt}$ is the log odds ratio for treatment *t* relative to the baseline treatment *b* which is assumed normally distributed with a mean $d_{bt}$ and between-study variance $\tau ^2$. It is assumed that there is consistency in the model such that $d_{bt}=d_{Ct}-d_{Cb}$, where *C* is the control or reference treatment. When conducting NMA using a Bayesian framework, priors for $\mu _{ib}$, $d_{bt}$ and $\tau$ are required.

To include studies investigating mixed biomarker populations, the standard NMA model described in (25) is adapted to downweight studies investigating biomarker mixed populations compared to studies investigating the biomarker subgroup of interest. Downweighting is achieved by inflating the variance of studies with mixed populations:

26 $$\begin{aligned} r_{it} \sim Bin(p_{it}, n_{it}) \\ logit(p_{it})= \left\{ \begin{array}{lr} \mu _{ib} & \text {for}\ t=b\\ \mu _{ib} + \delta _{ibt} & \text {for}\ t\neq b\\ \end{array}\right. \\ \delta _{ibt} \sim N \left( d_{bt}, \frac{\tau ^2}{w_{i}} \right) \sim N \left( d_{Ct}-d_{Cb}, \frac{\tau ^2}{w_{i}} \right) \end{aligned}$$

where $0<w_{i}<1$ is used to down-weight studies with mixed patient populations. Proctor et al. [31] suggest investigating two different choices for $w_{i}$:

1. The proportion of biomarker-positive patients per study, $x_{i}$ is assigned to $w_{i}$ such that studies with a higher proportion of biomarker-positive patients contribute more evidence than studies with only a few biomarker-positive patients and studies including only biomarker-positive patients ($x_{i}=1$) are included without down-weighting ($w_{i}=1$).
2. The weight of studies with $x_{i}<1$ is decreased by assigning $w_{i}$ a uniform prior distribution which depends on the assumed trust in the evidence of mixed population studies evaluated at varying levels of trust in the mixed populations’ data:
  $$w_{i} \sim Unif(0,0.3)$$
  $$w_{i} \sim Unif(0.3, 0.7)$$
  $$w_{i} \sim Unif(0.7, 1)$$

The potential key advantage of this approach is that it does not necessarily require any information about the biomarker subgroups. For example, to conduct the *Wheaton et al.* method, it is necessary to extract information on the percentage of biomarker-positive patients in the study investigating the mixed population. In *Enriching Through Weighting*, it is possible to assign a uniform prior distribution based on the level of “trust" the researcher has in the data from mixed populations. However, Proctor et al. [31], in their simulation study, found that this approach produced biased results in several scenarios and, therefore, could not be recommended. Furthermore, they found that the *Enriching Through Weighting* approach achieved the lowest bias and highest precision when little weight was assigned to studies conducted in mixed populations. Finally, this model does not presently extend to cases where there is more than one relevant biomarker of interest.

### Informative prior

The *Informative Prior* approach was originally presented by Efthimiou et al. [30] and adapted by Proctor et al. [31] for the synthesis of studies investigating different biomarker groups. Proctor et al. do not provide code to implement this model however, this should be simple to implement as it only requires a standard MA or NMA to be conducted, first on the mixed population and then on the population of interest using informative priors obtained from the analysis of the mixed population. As with the *Enriching Through Weighting* approach, there are studies investigating the biomarker subgroup of interest, biomarker-positive patients, and studies where patients are biomarker-mixed. The *Informative Prior* approach is conducted in two stages:

1. A NMA of studies with a mixed patient population is conducted to calculate a mean relative treatment effect, $d_{bt}^{mix}$, and variance, $\sigma _{bt}^{{mix}^2}$. This is just a standard NMA (as in Eq. (25) applied to studies investigating biomarker-mixed patients only).
2. The mean treatment effect and variance estimated from the biomarker-mixed studies is used to define a prior distribution for the treatment effect in a meta-analysis synthesising studies investigating only biomarker-positive patients. To account for the fact that the populations in stage 1 and stage 2 of the analysis are systematically different, the variance of the prior distribution can be inflated by a pre-defined factor *w*, to ensure that the prior in stage 2 is wider than the posterior from stage 1 if systematically differing populations are expected. The choice of $0<w<1$ depends on the assumed agreement between evidence from biomarker-positive studies and biomarker-mixed studies. Proctor et al. [31] assumed moderate agreement with a distribution of $w \sim Unif(0.3, 0.7)$.

A disadvantage of this model, similar to the *Enriching Through Weighting* approach is that the model currently does not extend to more than one biomarker of interest. Furthermore, it is possible that simply inflating the variance of the informative prior distribution is insufficient when incorporating studies from a mixed population with studies in a biomarker-positive population as not only will the variance differ, but the point estimate is also likely to differ. This method could be further adapted to include a systematic difference term to account for potential differences in point estimates between the two populations.

### Network meta-interpolation

Harari et al. [33] identified that while there are several methods using IPD to adjust for effect modification in NMA, these methods disregard subgroup information from aggregated data, thus wasting potentially valuable information. Therefore, they propose a network meta-interpolation (NMI) to balance patient populations and use regression to relate outcomes and effect modifiers. Harari et al. provide code in R to implement all models described in their paper.

To conduct NMI it is necessary to have:

1. A connected treatment network
2. Dichotomous effect modifiers reported at least at the study-level
3. IPD capturing correlations between all effect modifiers or correlations known from the data
4. Treatment effect estimates and standard errors at study-level and both levels of all effect modifiers for all AD studies

Consider that there are two effect modifiers $X_{1}$ & $X_{2}$ and that no information on the distribution of either effect modifier is reported in the subgroup analyses with respect to the other.

The first step of NMI is to impute missing values for the two effect modifiers using the best linear unbiased predictor (BLUP):

27 $$\begin{aligned} X_{n}^{*}(X_{m}) = \hat{\rho }_{\bar{X}_{1},\bar{X}_{2}}\hat{\sigma }_{\bar{X}_{1}}\hat{\sigma }_{\bar{X}_{2}}(X_{i} - \bar{X}_{j}) + \bar{X}_{j}, 1 \le m < n \le 2 \end{aligned}$$

where $\hat{\rho }_{\bar{X}_{1},\bar{X}_{2}}$ is the Pearson correlation (known from literature or estimated using trial IPD), $\bar{X}_{1}$ & $\bar{X}_{2}$ are the mean proportions for the effect modifiers and $\hat{\sigma }_{\bar{X}_{1}}$ & $\hat{\sigma }_{\bar{X}_{2}}$ are their standard deviations.

The second step of NMI is to estimate the treatment effect and associated variance at specified values of the effect modifiers:

28 $$\begin{aligned} (\bar{x}_{1}, \bar{x}_{2}) := (P(X_{1}=1), P(X_{2}=1)) \end{aligned}$$

The first two steps are applied to every study in the NMA. The final step is to apply a standard NMA to these treatment effects and associated variances.

Harari et al. [33] acknowledge that detailed data are required to utilise the NMI method and provide several practical solutions for the scenario where subgroup analyses are not reported. If the head-to-head comparison is not reported in any other study in the network, it can be assumed that the effect modifiers under consideration do not modify the relative treatment effect. However, if the head-to-head comparison is conducted in another study in the network, and all relevant subgroup analyses have been conducted, it is possible to borrow information about how different covariates modify the relative treatment effect while offsetting the treatment effect estimate according to a study-specific baseline.

An advantage of this approach is that it can be used to incorporate multiple biomarkers of interest and can be adapted to include categorical variables by recoding them into multiple binary variables. There are several disadvantages of this approach including the assumption of an identical correlation structure between effect modifiers across all studies and the requirement of availability of data at subgroup level for all studies and effect modifier levels.

### Proctor et al. method

Proctor et al. [34] developed an NMA model to synthesise IPD and AD evidence for targeted therapies on patient subgroups and for non-targeted therapies on a mixed patient population with regard to a special biomarker status, when there are no separate treatment effect estimates for biomarker-positive patients available. Code is provided in the supplementary material of the manuscript to implement the models described.

Proctor et al. [34] consider a network with three treatments. These are a control therapy (*C*), a standard therapy (*S*) and an experimental therapy which is only available for biomarker-positive patients ($$E_{+})$$. The authors consider that there is IPD available from trials investigating $$E_{+}$$ vs *C* and that AD is available from trials investigating *C* vs *S*. The authors are interested in the treatment effect of the target therapy ($$E_{+}$$) compared to the standard therapy (*S*) in biomarker-positive patients based on all direct and indirect evidence.

The first part of the model is to model the IPD. Consider that $Y_{ijC}$ and $Y_{ijE_{+}}$ are the binary responses of the $j^{th}$ participant in treatment arm *C* and $E_{+}$ respectively in the $i^{th}$ study and they follow a Bernoulli distribution with the probability of event of $p_{ijC}$ and $p_{ijE_{+}}$ respectively:

29 $$\begin{aligned} Y_{ijC} \sim Bernoulli(p_{ijC}) \end{aligned}$$

30 $$\begin{aligned} Y_{ijE_{+}} \sim Bernoulli(p_{ijE_{+}}) \end{aligned}$$

Standard logistic regression is fitted to each participant *j* of *ith* study where $\mu _{iC}^{IPD}$ represents the log odds for the control group *C* in study *i* and $\delta _{iCE_{+}}$ is the log odds ratio for treatment $E_{+}$ related to control *C*:

31 $$\begin{aligned} logit(p_{ijE_{+}}) = \mu _{iC}^{IPD} + \delta _{iCE_{+}} \end{aligned}$$

32 $$\begin{aligned} \delta _{iCE_{+}} \sim N(d_{CE_{+}}, \tau ^2) \end{aligned}$$

This model is conducted using a Bayesian approach, thus appropriate prior distributions must be used.

The second part of the model is to model the studies where only AD are available. Proctor et al. [34] do this via a meta-regression random-effects model which can model arm-based responses dependent on the proportion of biomarker-positive patients in each study:

33 $$\begin{aligned} r_{iC} \sim Bin(p_{iC}, n_{iC}) \\ r_{iS} \sim Bin(p_{iS}, n_{iS}) \\ logit(p_{iC}) = \mu _{iC}^{AD} \\ logit(p_{iS}) = \mu _{iC}^{AD} + \delta _{iCS} + \beta _{CS}^{B}X_{i} \\ \delta _{iCS} \sim N(d_{CS}, \tau ^{2}) \end{aligned}$$

The term $\beta _{CS}^{B}X_{i}$ represents a study-level specific covariate regression term for *S* relative to *C* for each study *i* where the covariate $X_{i}$ represents the mean percentage of biomarker-positive patients in study *i*. Once again prior distributions are required here.

The final part of the model combines the estimates of treatment effects for standard treatment (*S*) and the new targeted treatment ($E_{+}$) versus the control treatment (*C*) to obtain an indirect effect estimate for *S* versus $E_{+}$. To do this, consistency in the network must be assumed:

34 $$\begin{aligned} \delta _{iE_{+}S} \sim N(d_{E_{+}S}, \tau ^2) \sim N(d_{CS}-d_{CE{+}} + \beta _{CS}^{B}X_{i}, \tau ^2) \end{aligned}$$

This method developed by Proctor et al. [34] has the ability to estimate treatment effects for a patient subgroup based on direct and indirect evidence integrating comparisons of targeted and non-targeted therapies. The model utilises meta-regression and the proportion of biomarker-positive and biomarker-negative patients meaning that no assumption is required about the treatment effects in biomarker subgroups. However, the use of meta-regression in the AD part of the model introduces limitations to this approach such as the potential for aggregation bias and high variance when there are few studies included. Furthermore, at present this model has not been extended to scenarios where more than one biomarker group is of interest.

### Umemneku-Chikere et al. method

Umemneku-Chikere et al. [35] adapt the method developed by Proctor et al. [34] to allow for synthesis of AD and IPD from trials where the outcome is measured as time-to-event. Umemneku-Chikere et al. provide code to implement these models in the supplementary material of the manuscript describing these methods. As in Proctor et al. [34] in the first stage, IPD is modelled. For time-to-event outcomes Umemneku-Chikere et al. [35] model time-to-event data assuming a Weibull distribution. The log hazard ($\lambda _{ij}$) depends on the biomarker status of the patient ($X_{ij}$) and the treatment they receive ($T_{ij}$):

35 $$\begin{aligned} log(\lambda _{ij}) = \mu _{-ve,i} + \beta _{ij}X_{ij} + \delta _{-ve,i,tb}T_{ij} + \Delta _{i,tb}X_{ij}*T_{ij} \end{aligned}$$

where $\mu _{-ve,i}$ and $\delta _{-ve,i,tb}$ are the baseline treatment effect and relative treatment effect (log hazard ratio of treatment *b* vs treatment *t*) respectively, in study *i* for biomarker-negative patients. For biomarker-positive patients, baseline and relative treatment effects are $\mu _{+ve,i}$ and $\delta _{+ve,i,tb}$, such that the differences in the baseline and relative treatment effects between biomarker groups are:

36 $$\begin{aligned} \beta _{ij} = \mu _{+ve,i} - \mu _{-ve,i} \end{aligned}$$

37 $$\begin{aligned} \Delta _{i,tb} = \delta _{+ve,i,tb} - \delta _{-ve,i,tb} \end{aligned}$$

Relative treatment effects are assumed exchangeable within each biomarker subgroup and treatment contrast:

38 $$\begin{aligned} \delta _{-ve,j,tb} \sim N(md_{-ve,tb}, \tau ^2)\end{aligned}$$

39 $$\begin{aligned} \delta _{+ve,j,tb} \sim N(md_{+ve,tb}, \tau ^2) \end{aligned}$$

As is usual in NMA, the mean effects ($md_{-ve,tb}$ and $md_{+ve,tb}$) within each treatment contrast are assumed to satisfy the consistency assumption. Umemneku-Chikere et al. [35] develop this model in a Bayesian framework and recommend using $\gamma _{j} \sim Gamma(1, 0.01)$, $\mu _{+ve,j} \sim N(0,100)$ and $\mu _{-ve,j} \sim N(0,100)$ as vague prior distributions.

Also as in Proctor et al. [34], the second stage is to model studies where only AD are available:

40 $$\begin{aligned} \hat{\delta }_{i,tb} \sim N(\delta _{i,tb}, \sigma _{i}^2) \\ \delta _{i,tb} \sim N(md_{i,tb}, \tau ^2) \\ md_{i,tb} = d_{-ve,b} - d_{-ve,t} + (\bar{\beta }_{b} - \bar{\beta }_{t}) * z_{i} \end{aligned}$$

where $\hat{\delta }_{i,tb}$ and $\sigma _{i}^2$ is the observed treatment effect and variance of this treatment effect for each study *i*. The observed treatment effect in study *i* is assumed to come from a normal distribution with a mean “true" underlying treatment effect ($\delta _{i,tb}$) and within-study variance ($\sigma _{i}^2$). The true treatment effects are assumed to follow a common distribution in each treatment contrast *tb* where the basic parameters are $d_{-ve,b}$ and $d_{-ve,t}$ and correspond effects of treatments t and b in the biomarker-negative group, as in the IPD section of the analysis. $\bar{\beta }_{b}$ and $\bar{\beta _{t}}$ are the study-level meta-regression coefficients corresponding to the proportion of biomarker-positive participants in study *i* ($z_{i}$). Therefore, the basic parameters for the biomarker-positive subgroup are given by:

41 $$\begin{aligned} d_{+ve,t} = d_{-ve,t} + \bar{\beta }_{t} \end{aligned}$$

42 $$\begin{aligned} d_{+ve,b} = d_{-ve,b} + \bar{\beta }_{b} \end{aligned}$$

The authors conduct this model using a Bayesian approach and using the prior distributions $\bar{\beta }_{t,b} \sim N(0,100)$.

The final part of the model combines IPD and AD through the shared parameters $d_{-ve,b}$, $d_{+ve,b}$, $d_{-ve,t}$ and $d_{+ive,t}$. Prior distributions are used:

43 $$\begin{aligned} d_{-ve,t,b} \sim N(0,100) \\ \tau \sim Unif(0,2) \end{aligned}$$

This model extends the method described by Proctor et al. [34] to synthesise time-to-event data. Once again this model utilises meta-regression and the proportion of biomarker-positive and biomarker-negative patients meaning that no assumption is required about treatment effects in biomarker subgroups. However, the use of meta-regression can introduce limitations as previously described. In addition, as in Proctor et al. [34] this model cannot currently include more than one biomarker subgroup of interest.

## Comparison of methods

### Data

**Fig. 2:**
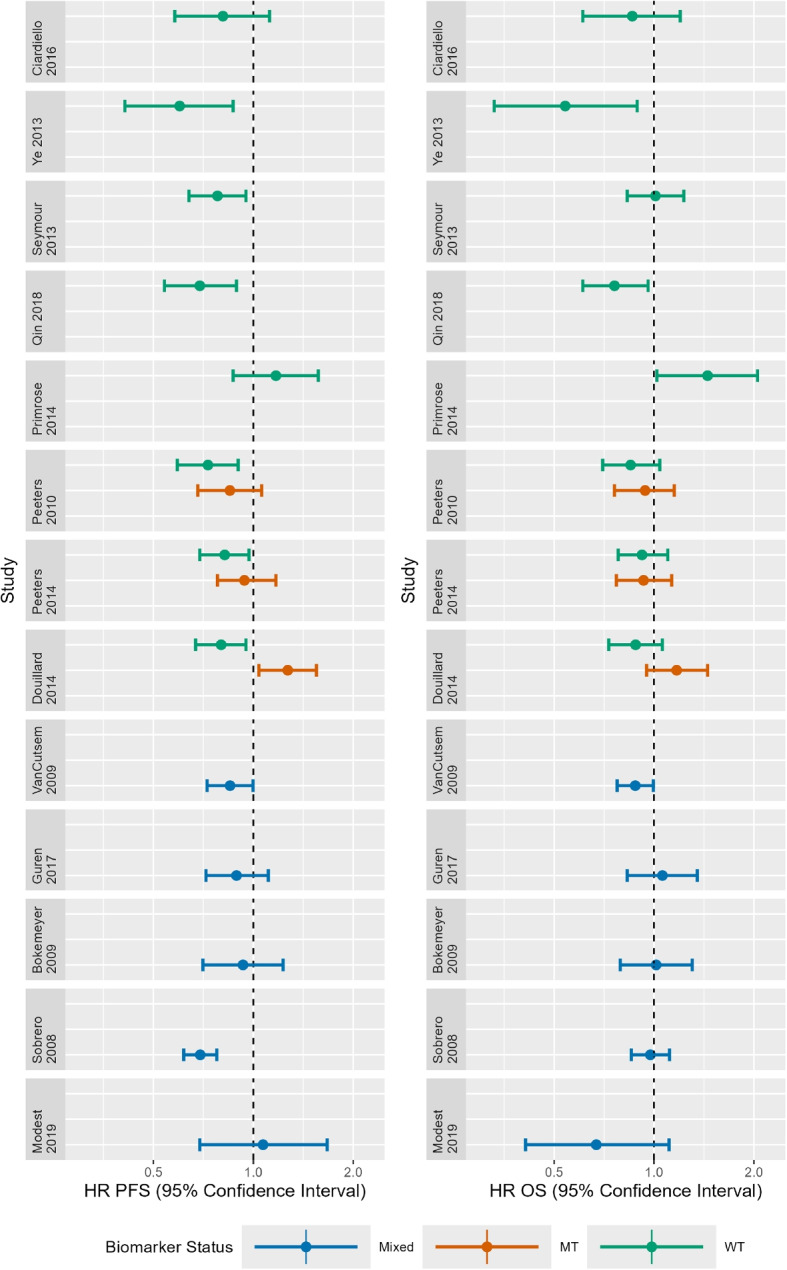
Observed hazard ratios and 95% confidence intervals on progression-free survival and overall survival

We illustrate how to implement several of the identified methods by revisiting an example of anti-EGFR therapies for treatment of metastatic colorectal cancer (mCRC) which has been previously described in more detail [26]. Over time evidence has emerged to suggest that anti-EGFR therapies are effective in patients with wild-type (WT) KRAS biomarker status but ineffective in patients with mutant-type (MT) KRAS biomarker status. Anti-EGFR therapies have been evaluated in trials with different designs and varying KRAS biomarker populations. The data used in this analysis were obtained from 13 RCTs and studies report hazard ratios (HRs) and corresponding confidence intervals (CIs) on progression-free survival (PFS) and overall survival (OS) which were converted into logHRs and corresponding standard errors for analysis. The data are represented graphically in Fig. 2.

### Methods

Seven methods are applied to the illustrative example in mCRC. First a standard random-effects meta-analysis is applied to treatment effects obtained from the biomarker subgroup of interest alone. This shall henceforth be referred to as the *Stand Alone* approach. Second a standard random-effects meta-analysis is applied to treatment effects obtained from all treatment effects regardless of biomarker population. This shall henceforth be referred to as the *Naive* approach. Third a *Meta-regression* is applied. These three standard methods were chosen to apply to the illustrative example as they are well-established methods which are frequently used and can be applied to AD in pairwise meta-analysis.

In addition, four of the eight identified methods are applied to the illustrative example. These are the *Wheaton et al.*, *Extended Within Trial Framework*, *Enriching Through Weighting* and *Informative Prior* approaches. These approaches were selected as they (a) had available code to conduct the models, (b) can be applied using AD only and (c) are methods for or can be adapted for pairwise meta-analysis. *NMI*, *Proctor et al.* and *Umemneku-Chikere et al.* were not included as they are methods for NMA and required information from IPD which was not available for the illustrative example. We were unable to include *LMM* in the model comparison as there was not code available to implement the model incorporating mixed biomarker populations.

Unless otherwise specified, models were fitted using Stan via the **RStan** package [36] using 3 chains with 4000 iterations (1000 warmup) per chain and vague prior distributions for model parameters. Model convergence was assessed via visual inspection of trace, density and autocorrelation plots generated using the Shiny application **shinystan** [37]. The code used to conduct this analysis is available in the supplementary material.

#### Stand alone

In the *Stand Alone* analysis, a standard Bayesian random-effects meta-analysis is applied to treatment effects on PFS and OS reported in KRAS WT populations only.

#### Naive

In the *Naive* analysis, a standard Bayesian random-effects meta-analysis is applied to treatment effects on PFS and OS reported in KRAS WT, MT and mixed populations.

#### Meta-regression

In the *Meta-regression* analysis, the method is applied in two ways. Ideally in a meta-regression the pooled treatment effect and proportion of biomarker-positive patients will be available for every study. However, in this dataset some studies do not report the pooled effect and only report subgroup effects separately for KRAS WT and KRAS MT patients. Therefore, there are two options for how to conduct the meta-analysis:

- Include **only** KRAS WT subgroup for studies reporting subgroup effects
- Include **both** KRAS WT and KRAS MT subgroups for studies reporting subgroup effects

Where a study reported the proportion of MT patients for a subset of the biomarker-mixed population, this proportion was directly used in the meta-regression. Where a study did not report KRAS status for any patients, the proportion is assumed to be 0.42 (the mean of the beta distribution used in *Wheaton et al.* analysis).

#### Wheaton et al.

In the *Wheaton et al.* analysis, the method is applied to treatment effects on PFS and OS reported in KRAS WT, MT and Mixed populations. Where a study reported the proportion of MT patients for a subset of the biomarker-mixed population (Van Cutsem 2009, Guren 2017, Bokemeyer 2009), informative beta prior distributions for the proportions were constructed using the method of moments approach. Where a study did not report KRAS status for any patients (Sobrero 2008 and Modest 2019) beta prior distributions were constructed using information on the prevalence of KRAS mutations in mCRC patients obtained from the literature suggesting that frequency of mutations is between 30% and 54% [38]. Details of how the prior distributions were constructed is available in Appendix 5.

#### Extended within-trial framework

In the *Extended Within-Trial Framework* analysis, the method is applied to treatment effects on PFS and OS reported in KRAS WT and MT populations. It should be noted that this model cannot include information from studies conducted in mixed populations without subgroup analysis. Therefore, the five mixed studies are not included in this analysis. The model is fitted using the **metafloat** [29] package in Stata assuming unstructured random-effects to allow for a different heterogeneity variance to be estimated within each subgroup.

#### Enriching through weighting

In the *Enriching Through Weighting* analysis, the method is applied to treatment effects on PFS and OS reported in KRAS WT and Mixed populations. There are various choices for downweighting the treatment effects obtained from the biomarker mixed populations. Following the suggestions by Proctor et al. [31] the model is fitted using the proportion of KRAS WT patients in each study. As for *Meta-regression*, where a study reported the proportion of KRAS WT patients for a subset of the biomarker-mixed population, this proportion was used in the analysis. Where a study did not report KRAS WT status for any patients, the proportion was assumed to be 0.58. However, unlike in Proctor et al. [31] uniform prior distributions are not used as uniform priors are not recommended to use within Stan as it hinders model convergence. Therefore, normal distributions (truncated at lower value of zero and upper value of 1) were used:

- $$w_{i} \sim N(0.15, 0.075^2)$$
- $$w_{i} \sim N(0.5, 0.10^2)$$
- $$w_{i} \sim N(0.85, 0.075^2)$$

#### Informative prior

In the *Informative Prior* analysis, the method is applied to treatment effects on PFS and OS reported in KRAS WT and Mixed populations. Proctor et al. [31] suggest that downweighting can be applied to the informative prior based on the level of “trust" in the data. However, in this example the informative prior obtained from the analysis of mixed biomarker data is used without inflating the variance to downweight this information.

### Results

**Fig. 3:**
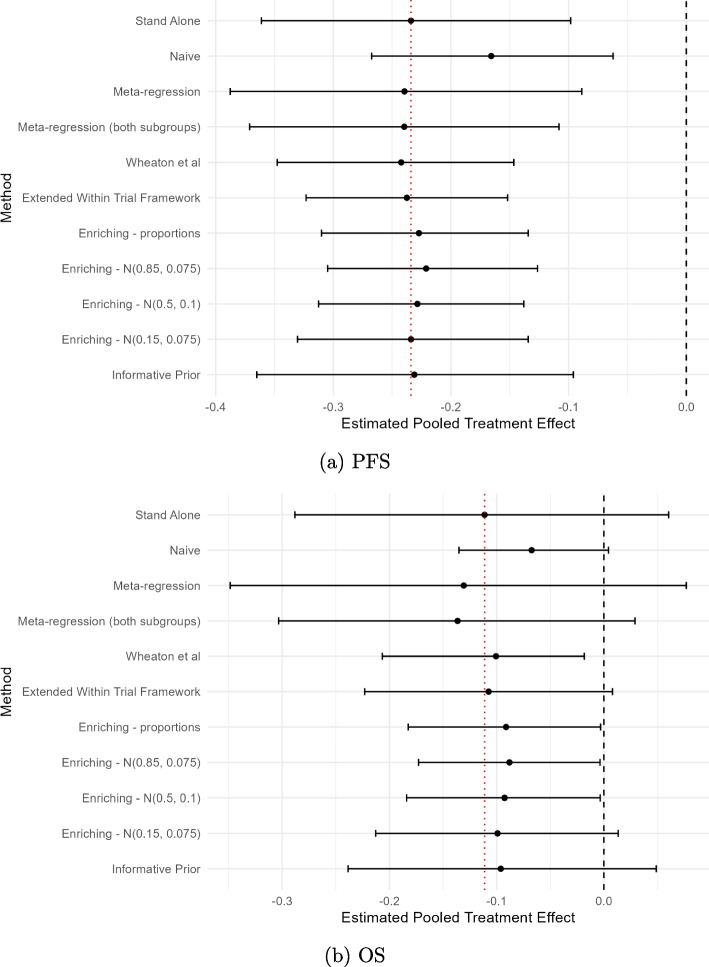
Comparison of estimated pooled treatment effect (logHRs) in KRAS WT biomarker subgroup for PFS (**a**) and OS (**b**) across methods

Figure 3 shows the results of the application of the models to the PFS and OS data of the illustrative example. It can be observed that all models estimated a treatment benefit for anti-EGFR therapies compared to chemotherapy. With the exception of the *Naive* approach, the pooled treatment effect estimates were reasonably consistent, being estimated within a range of 0.021 and 0.048 for PFS and OS respectively. Furthermore, with the exception of the *Naive* approach, the pooled treatment effect estimates from each of the methods were reasonably close to the pooled treatment effect estimate obtained from the *Stand Alone* model. For the PFS outcome, the largest deviation of the mean effect point estimate from the mean effect estimated by the *Stand Alone* model was 0.013 from the *Enriching Through Weighting* model with *N*(0.85, 0.075) prior. For the OS outcome, the largest deviation of the mean effect point estimate from the mean effect estimated by the *Stand Alone* model was 0.025 from the *Meta-regression* model using both subgroups. With the exception of *Meta-regression* and *Informative Prior* methods, all methods estimated narrower 95% CrI (or CI) intervals compared to the 95% CrI estimated from the *Stand Alone* method. Excluding the *Naive* analysis, the *Extended Within Trial Framework* and *Enriching Through Weighting* approaches resulted in the largest improvements in uncertainty for PFS and OS respectively compared to the *Stand Alone* model. Information on the percentage change in CrI width is available for all models in Table 4 in Appendix 6.

## Discussion

The development of precision medicine means that increasingly, treatments are developed and evaluated in trials of mixed designs and with mixed populations. For example, treatments might initially be developed and evaluated in biomarker-mixed populations resulting in trials on the mixed population with no subgroup analysis. However, biomarkers are then subsequently evaluated in exploratory subgroup analyses resulting in trials on mixed populations reporting subgroup analysis. If biomarker status is found to be predictive of treatment effect such that only biomarker-positive patients benefit from treatment, later trials are likely to be conducted in biomarker-positive populations only. This heterogeneity in trial design may pose a difficulty when conducting evidence synthesis using standard meta-analytic tools. We sought to identify efficient methods that allow synthesis of data from mixed populations and provided descriptions of these methods.

We identified eight methods for combining data from mixed populations in a single evidence synthesis. Three of these methods (*Wheaton et al.*, *LMM* and *Extended Within Trial Framework*) proposed using AD to combine studies with mixed populations in a single pairwise meta-analysis. However, it is unlikely that subgroup treatment effects and treatment-covariate interactions will be available from published papers for every study in the meta-analysis and thus in practical terms it is likely that IPD will be required to utilise the *Extended Within Trial Framework*. Three of the eight identified methods (*Enriching Through Weighting*, *Informative Prior* and *NMI*) described techniques for NMA using only AD. However, *Enriching Through Weighting* and *Informative Prior* approaches described in the context of mixed biomarker populations by Proctor et al. [31] could easily be adapted and applied in the pairwise meta-analysis setting. Furthermore, while Harari et al. [33] propose that *NMI* can be implemented in the absence of IPD by using an external estimate of the correlation between effect modifiers needed to populate their model, it would be preferable to obtain IPD from at least one study in the meta-analysis to allow correlation to be estimated directly. The final two identified methods (*Proctor et al.* for binary data and *Umemneku-Chikere et al.* method for time-to-event data) are applicable for NMA using both AD and IPD.

When assessing these methods various advantages and limitations were identified. Generally, methods which are conducted using AD alone are more straightforward to implement as AD are more likely to be accessible through trial reports. However, by using only AD these methods make assumptions which are not necessarily reasonable and can lead to poor performance of the methods. For example, *Wheaton et al.* assumes a linear relationship between treatment effects in the biomarker-positive subgroup, the proportion of biomarker-positive patients in the study and the treatment effects in the biomarker-mixed population when it is known that this relationship does not exactly hold for non-collapsible measures of treatment effect. Furthermore, *Proctor et al.* and *Umemneku-Chikere et al.* utilise meta-regression to include AD which has low power to detect treatment-covariate interactions and can be subject to aggregation bias. Methods which are not subject to these issues such as the *Extended Within Trial Framework* are preferable for their statistical qualities. However, such methods are practically more difficult to implement as gaining access to IPD for all relevant studies is often time consuming and frequently not possible. As is true in general in the field of meta-analysis, for these methods there is a trade-off between using AD for speed and convenience and IPD for the highest quality analysis.

Each of the methods papers identified were input to SpiderCite on the The Evidence Review Accelerator website to identify any papers which cited the methodology papers [39]. The only method cited in an applied paper was the *Extended Within Trial Framework* which was utilised in a systematic review and meta-analysis of IPD from RCTs investigating which patients with metastatic hormone-sensitive prostate cancer benefit from docetaxel [40]. While no other approaches were identified via SpiderCite, the *Stand Alone* and *Naive* approaches are often used in the literature. For example, Umemneku-Chikere et al. [41] conducted a NMA to investigate the effectiveness of therapies in HER2-positive advanced breast cancer in hormone receptor subgroups. This paper utilised both the *Naive* approach, by pooling all data in NMA, and the *Stand Alone* approach, by investigating hormone receptors in a subgroup analysis. The authors comment that subgroup analysis estimates were highly uncertain due to the limited reporting of subgroup analyses in RCTs. Novel methods discussed in this review will hopefully facilitate improved conclusions in future studies on synthesis of evidence in subgroups of populations. There is a need for more dissemination of complex methods such as those described in this review to increase application of these methods to real world problems.

When investigating these methods it is interesting to observe how the problem is framed. In three of the identified methods (*Wheaton et al.*, *Enriching Through Weighting* and *Informative Prior*) the problem is framed as if only one biomarker subgroup was of interest. However, other methods, such as *Extended Within Trial Framework*, imply that it is not only important to utilise all relevant information to estimate treatment effects in both subgroups but that it is also crucial to estimate the treatment-biomarker interaction. Which of these methods is most appropriate will depend on the decision-making problem. For example, consider that over the development process for a new treatment it became clear that the treatment is only effective in patients in the biomarker-positive subgroup leading to the treatment subsequently being evaluated in enrichment trials (in biomarker-positive patients alone). Consequently, there would be trials conducted in biomarker-mixed populations with and without subgroup analysis and trials conducted in biomarker-positive patients only. When the treatment is assessed by a health technology assessment (HTA) agency, decision-making will be based on the treatment being given to biomarker-positive patients only. In this context, only clinical and cost-effectiveness estimates for the biomarker-positive subgroup are relevant. Therefore, in this scenario the *Wheaton et al.*, *Enriching Through Weighting* and the *Informative Prior* approach are potentially more beneficial. However, if during drug development there is reason to believe that a treatment is more beneficial in one biomarker subgroup, exploratory analysis should be conducted to determine if there is an interaction between treatment and biomarker status and determine treatment effects in the biomarker subgroups. In this scenario looking at a single subgroup is insufficient as while the treatment effect may be more beneficial in one biomarker-subgroup, this does not necessarily mean that there is no treatment effect in the other biomarker-subgroup. Therefore, in this case the *Extended Within Trial Framework* is a more beneficial approach.

Finally, there are several limitations of this review of methods. First, the search was restricted to records identified from the PubMed database and thus restricted to methods available in the field of life sciences and medicine. However, it is possible that relevant methods might be available outside these fields and thus restriction to the PubMed database will result in potentially relevant methods having been overlooked. Future research should consider the expansion of this review to additional databases outside the life sciences and medicine field. Second, several papers have been published since the publication search took place including an article by Evrenoglou et al. [42] regarding sharing information across patient subgroups to draw conclusions from sparse treatment networks. Therefore, future research should consider regularly updating this review in order to remain on top of developments in the field. Third, we have applied several of the methods to an illustrative example in mCRC, however, the application was limited to *Stand Alone*, *Naive*, *Meta-regression*, *Wheaton et al.*, *Extended Within Trial Framework*, *Enriching Through Weighting* and *Informative Prior* approaches. The remaining identified methods could not be applied as; (a) there was no code available to conduct the models, (b) the models required IPD or (c) the methods could not be adapted for pairwise meta-analysis. Future research could identify a NMA with IPD available for all trials such that all methods can be applied and compared. Furthermore, to not only apply these methods but evaluate their properties, a simulation study could be conducted.

## Conclusions

In conclusion, this paper has provided a background description of methods for meta-analysis and identified various methods which have been proposed to synthesise evidence from mixed populations. We have described the main assumptions, advantages and limitations associated with each method. As in the meta-analysis literature generally, methods to synthesise mixed populations can be split into methods using AD, methods using IPD and methods utilising both AD and IPD with methods utilising IPD achieving superior statistical properties at the expense of increased difficulty in accessing all relevant data. However, the methods can also be considered based on the context of the decision problem they are addressing with some methods focusing on estimating the treatment effect in only the subgroup of interest and some methods focusing on the estimation of treatment effects in both subgroups. Therefore, when synthesising data from trials with mixed populations it is important to consider context in order to select the most appropriate modelling framework.

## Supplementary Information

Supplementary Material 1.

## Data Availability

Data and code to conduct the comparison of methods is available in the supplementary material.

## Appendix 1: meta-analysis - binomially distributed outcomes

### Fixed-effect

For binomially distributed outcomes, a fixed-effect meta-analysis for normally distributed outcomes can be utilised using data such as log odds ratios (logOR). However, the assumption of normality can be avoided by using the exact binomial distribution to model the data:

44 $$\begin{aligned} r_{Ai} \sim Bin(p_{Ai}, n_{Ai}) \\ r_{Bi} \sim Bin(p_{Bi}, n_{Bi}) \\ logit(p_{Ai}) = \mu _{i} \\ logit(p_{Bi}) = \mu _{i} + \delta \end{aligned}$$

where $n_{Ai}$ and $n_{Bi}$ are the number of participants and $p_{Ai}$ and $p_{Bi}$ are the probabilities of event in arms A and B of each study *i*. $\mu _{i}$ is the baseline treatment effect in arm A of the study and $\delta$ is the fixed treatment effect. When conducting a fixed-effect meta-analysis for binomially distributed outcomes in a Bayesian framework, prior distributions are required for $\mu _{i}$ and $\delta$

### Random-effects

Similarly, random-effects meta-analysis can be conducted for binomially distributed outcomes by including a random-effect on the treatment effect:

45 $$\begin{aligned} r_{Ai} \sim Bin(p_{Ai}, n_{Ai}) \\ r_{Bi} \sim Bin(p_{Bi}, n_{Bi}) \\ logit(p_{Ai}) = \mu _{i} \\ logit(p_{Bi}) = \mu _{i} + \delta _{i} \\ \delta _{i} \sim N(d, \tau ^2) \end{aligned}$$

When random-effects meta-analysis for binomially distributed outcomes is conducted in a Bayesian framework, prior distributions are required on $\mu _{i}$, *d* and $\tau$ parameters.

## Appendix 2: network meta-analysis (NMA)

Decision-makers often want to compare more than two treatments in order to answer questions such as “which treatment is most effective overall". Network meta-analysis (NMA) is an extension of pairwise meta-analysis which allows the synthesis of data on more than two interventions.

### Random-effects

#### Normally distributed data

For data which can be assumed normally distributed a random-effects NMA is defined as:

46 $$\begin{aligned} Y_{ibk} \sim N(\delta _{ibk}, \sigma _{i}^2) \\ \delta _{ibk} \sim N(d_{bk}, \tau _{bk}^2) \end{aligned}$$

where $Y_{ibk}$ indicates the observed treatment effect of treatment *k* versus treatment *b* in study *i* which is assumed to be normally distributed and estimates the underlying true treatment effects $\delta _{ibk}$. The true treatment effects follow a normal distribution with a mean which is represented in terms of indirect effects:

47 $$\begin{aligned} d_{bk} = d_{Ck} - d_{Cb} \end{aligned}$$

where $d_{Ck}$ ($d_{Cb}$) are treatment effects in each arm *k* (*b*) relative to the reference treatment C.

When conducting NMA for normally distributed data using a Bayesian framework, priors for $d_{Ck}$ and $\tau _{bk}$ are required.

#### Binomially distributed data

Based on notation from Dias et al. [32], a random-effects NMA for a binary outcome is defined as:

48 $$\begin{aligned} r_{ik} \sim Bin(p_{ik}, n_{ik}) \\ logit(p_{ik})= \left\{ \begin{array}{lr} \mu _{ib} & \text {for}\ k=b\\ \mu _{ib} + \delta _{ibk} & \text {for}\ k\neq b\\ \end{array}\right. \\ \delta _{ibk} \sim N(d_{bk}, \tau ^2) \end{aligned}$$

where the indices *i* and *k* indicate study and treatment arm respectively. $r_{ik}$ denotes the number of observed events and $n_{ik}$ denotes the number of individuals per study arm. $\mu _{ib}$ are the log odds of an event for the baseline treatment and $\delta _{ibk}$ is the log odds ratio for treatment *k* relative to the baseline treatment *b* which is assumed normally distributed with a mean $d_{bk}$ and between-study variance $\tau ^2$. It is assumed that there is consistency in the model such that $d_{bk}=d_{Ck}-d_{Cb}$.

When conducting NMA using a Bayesian framework, priors for $\mu _{ib}$, $d_{bk}$ and $\tau$ are required.

### Network meta-regression (NMR)

Just as pairwise meta-analysis can be extended to meta-regression, NMA can be extended to network meta-regression (NMR) to investigate heterogeneity between treatment effects [43]. The linear predictor described in the set of Eqs. (48) can be extended to include a study-level covariate $z_{i}$:

49 $$\begin{aligned} logit(p_{ik}) = \mu _{i} + (\delta _{ibk} + \beta z_{i})I_{k\ne C} \end{aligned}$$

where $\beta$ denotes the interaction between covariate ($z_{i}$) and the study-level treatment effects ($\delta _{ibk}$). While the model in Eq. (49) assumes that the interaction effect is the same for all treatment contrasts, it is possible to further adapt the NMR model to assume that the interaction effects are independent (by stratifying the interaction term by the number of treatment comparisons) or that the interaction effects are not the same but follow a common distribution (by placing a random effect on the interaction).

## Appendix 3: combining AD and IPD

### Two-stage approach

Assume that there are $N_{IPD}$ trials which provide IPD and $N_{AD}$ trials providing only AD in the form of a treatment effect estimate and its variance such that the total number of trials in the meta-analysis is $N=N_{IPD}+N_{AD}$. The two-stage approach, as described in the One-stage and two-stage approaches section, can be easily adapted to incorporate studies which report only AD. In the first stage, a model would be fitted to the IPD from each trial separately to obtain estimates of the treatment effect and its variance and in the second stage, a fixed or random-effects meta-analysis would be applied to the treatment effect estimates and corresponding variances, obtained from IPD and AD trials, to estimate the pooled treatment effect and between-study variance.

### One-stage approach

The one-stage approach described in the One-stage and two-stage approaches section has also been extended by Riley et al. [44] to allow inclusion of studies providing IPD ($N_{IPD}$) and studies providing only AD ($N_{AD}$):

50 $$\begin{aligned} y_{ij}^* = D_{i}\alpha _{i} + \delta _{i}x_{ij} + e_{ij}^* \end{aligned}$$

51 $$\begin{aligned} \delta _{i} \sim N(d, \tau ^2) \end{aligned}$$

52 $$\begin{aligned} e_{ij}^* \sim N(0, V_{i}^*) \end{aligned}$$

where $D_{i}$ is a dummy variable to differentiate between studies providing IPD ($D_{i}=1$) and studies providing AD ($D_{i}=0$). For each trial providing IPD, $y_{ij}^*=y_{ij}$ and $V_{i}^*=\sigma _{i}^2$ as in the one-stage model described in Eqs. (6) & (7). For each trial providing AD, it is assumed that there is only one response such that $X_{i1}=1$, $y_{i1}^*=\hat{\delta }_{i}$ and $V_{i}^*=V(\hat{\delta }_{i})$. This ensures that only studies providing IPD can contribute to estimation of trial-level baselines ($\alpha _{i}$) and residual variances ($\sigma _{i}^2)$ but allows IPD and AD trials to estimate the pooled treatment effect (*d*) and between-study variance ($\tau ^2$).

## Appendix 4: search filters

(“Biostatistics”[Journal] OR

“Biometrics”[Journal] OR

“Biometrical journal. Biometrische Zeitschrift”[Journal] OR

“BMC medical research methodology”[Journal] OR

“Journal of clinical epidemiology”[Journal] OR

“Journal of the Royal Statistical Society. Series A, (Statistics in Society)”[Journal] OR

“Journal of the Royal Statistical Society. Series B, Statistical methodology”[Journal] OR

“Journal of the Royal Statistical Society. Series C, Applied statistics”[Journal] OR

“Research synthesis methods”[Journal] OR

“Statistics in medicine”[Journal] OR

“Statistical methods in medical research”[Journal] OR

“The annals of applied statistics”[Journal] OR

“Journal of the American Statistical Association”[Journal] OR

“Statistical science: a review journal of the Institute of Mathematical Statistics”[Journal]) AND

(biomarker*[Title/Abstract] OR

subgroup*[Title/Abstract]) AND

(meta-analysis[Title/Abstract] OR

“evidence synthesis”[Title/Abstract] OR

“synthesis”[Title/Abstract])

## Appendix 5: beta priors for proportions

### Van Cutsem 2009

**Table 1:** Number of patients in control and treatment arms in Van Cutsem 2009 for WT, MT and Mixed Analysis

	No. Control Patients	No. Treatment Patients	Total
WT	350	316	666
MT	183	214	397
WT + MT	533	530	1063
Mixed	599	599	1198

This paper reports KRAS status for 89% of the biomarker-mixed population. Where KRAS status is reported the proportion of MT patients $= \frac{397}{1063} = 0.373$.

Using variance formula:

$$\begin{aligned} Var(p) = \frac{p(1-p)}{n} \end{aligned}$$

Assuming $$p=0.373$$ and $$n=1063$$:

$$\begin{aligned} Var(p) = \frac{0.373(1-0.373}{1063} = 0.00022 \end{aligned}$$

Assuming a mean of 0.373 and a variance of 0.00022 a beta distribution can be constructed using method of moments calculation where:

$$\begin{aligned} mean&= m = \frac{\alpha }{\alpha + \beta } \\ var&= \frac{m(1-m)}{\alpha + \beta + 1} \end{aligned}$$

After method of moments calculation, this gives a beta prior distribution of:

$$\begin{aligned} p \sim Beta(396, 666) \end{aligned}$$

### Guren 2017

**Table 2:** Number of patients in control and treatment arms in Guren 2017 for WT, MT and Mixed analysis

	No. Control Patients	No. Treatment Patients	Total
WT	97	97	194
MT	58	72	130
WT + MT	155	169	324
Overall	185	194	379

This paper reports KRAS status for 86% of the biomarker-mixed population. Where KRAS status is reported the proportion MT patients $= 0.401$.

$$\begin{aligned} p&= \frac{130}{324} =0.401 \\ Var(p)&= \frac{0.401(1-0.401)}{324} = 0.000741 \end{aligned}$$

After method of moments calculation, this gives a beta prior distribution of:

$$\begin{aligned} p \sim Beta(129.6, 193.6) \end{aligned}$$

### Bokemeyer 2009

**Table 3:** Number of patients in control and treatment arms in Bokemeyer 2009 for WT, MT and Mixed analysis

	No. Control Patients	No. Treatment Patients	Total
WT	97	82	179
MT	59	77	136
WT + MT	156	159	315
Overall	168	169	337

This paper reports KRAS status for 94% of biomarker-mixed population. Where KRAS status is reported the proportion of MT patients $= 0.431$.

$$\begin{aligned} p&= \frac{136}{315} = 0.431 \\ Var(p)&= \frac{0.431(1-0.431)}{315} = 0.000779 \end{aligned}$$

After method of moments calculation, this gives a beta prior distribution of:

$$\begin{aligned} p \sim Beta(135.25, 178.56) \end{aligned}$$

### Sobrero 2008 and Modest 2019

These papers do not report the proportion of KRAS MT patients for any percentage of the population. Therefore, we define an informative beta prior distribution based on the prevalence of KRAS mutations in colorectal cancer.

In a study of 1018 cases of metastatic colorectal cancer, Neumann et al found KRAS mutations in 39.3% of patients which supported previous research reporting KRAS mutations in 30-54% of metastatic colorectal tumours.

We assume the prevalence of MT patients is normally distributed such that:

$$\begin{aligned} Range = mean \pm 2 \times SD \end{aligned}$$

Therefore, we assume the mean prevalence is 0.42 and the standard deviation is 0.06. We then used method of moments to obtain the following beta distribution:

$$\begin{aligned} p \sim Beta(28, 38.67) \end{aligned}$$

This beta prior distribution is used for mixed studies when the proportion of MT KRAS patients is unknown.

## Appendix 6: change in uncertainty

**Table 4:** Percentage change in CrI width of estimated pooled treatment effect compared to *Stand Alone* method

	Percentage Change CrI Width	
	PFS	OS
Meta-regression	14	22
Meta-regression (both subgroups)	0	-5
Wheaton et al	-23	-46
Extended Within Trial Framework	-35	-34
Enriching - proportions	-33	-48
Enriching - N(0.15, 0.075)	-25	-35
Enriching - N(0.5, 0.1)	-34	-48
Enriching - N(0.85, 0.075)	-32	-51
Informative Prior	2	-18

## References

[1] Tajik P, Zwinderman AH, Mol BW, Bossuyt PM. Trial Designs for Personalizing Cancer Care: A Systematic Review and ClassificationTrial Designs for Personalizing Cancer Care. Clin Cancer Res. 2013;19(17):4578–88. 23788580 10.1158/1078-0432.CCR-12-3722

[2] Twomey JD, Brahme NN, Zhang B. Drug-biomarker co-development in oncology-20 years and counting. Drug Resist Updat. 2017;30:48–62. 28363335 10.1016/j.drup.2017.02.002

[3] Amado RG, Wolf M, Peeters M, Van Cutsem E, Siena S, Freeman DJ, et al. Wild-type KRAS is required for panitumumab efficacy in patients with metastatic colorectal cancer. J Clin Oncol. 2008;26(10):1626–34. 18316791 10.1200/JCO.2007.14.7116

[4] Bokemeyer C, Bondarenko I, Makhson A, Hartmann JT, Aparicio J, De Braud F, et al. Fluorouracil leucovorin and oxaliplatin with and without cetuximab in the first-line treatment of metastatic colorectal cancer. J Clin Oncol. 2009;27(5):663–71. 19114683 10.1200/JCO.2008.20.8397

[5] Lievre A, Bachet JB, Le Corre D, Boige V, Landi B, Emile JF, et al. KRAS mutation status is predictive of response to cetuximab therapy in colorectal cancer. Cancer Res. 2006;66(8):3992–5. 16618717 10.1158/0008-5472.CAN-06-0191

[6] Sobrero AF, Maurel J, Fehrenbacher L, Scheithauer W, Abubakr YA, Lutz MP, et al. EPIC: phase III trial of cetuximab plus irinotecan after fluoropyrimidine and oxaliplatin failure in patients with metastatic colorectal cancer. J Clin Oncol. 2008;26(14):2311–9. 18390971 10.1200/JCO.2007.13.1193

[7] Van Cutsem E, Köhne CH, Láng I, Folprecht G, Nowacki MP, Cascinu S, et al. Cetuximab plus irinotecan, fluorouracil, and leucovorin as first-line treatment for metastatic colorectal cancer: updated analysis of overall survival according to tumor KRAS and BRAF mutation status. J Clin Oncol. 2011;29(15):2011–9. 21502544 10.1200/JCO.2010.33.5091

[8] Ciardiello F, Normanno N, Martinelli E, Troiani T, Pisconti S, Cardone C, et al. Cetuximab continuation after first progression in metastatic colorectal cancer (CAPRI-GOIM): a randomized phase II trial of FOLFOX plus cetuximab versus FOLFOX. Ann Oncol. 2016;27(6):1055–61. 27002107 10.1093/annonc/mdw136

[9] Deeks JJ, Higgins JP, Altman DG, Group CSM. Analysing data and undertaking meta-analyses. Cochrane Handb Syst Rev interventions. 2019;241–84.

[10] Riley RD, Tierney JF, Stewart LA. Individual participant data meta-analysis: a handbook for healthcare research. Oxford: Wiley; 2021.

[11] Nikolakopoulou A, Mavridis D, Salanti G. Demystifying fixed and random effects meta-analysis. BMJ Ment Health. 2014;17(2):53–7. 10.1136/eb-2014-101795 PMC13404554 24692250

[12] Mills EJ, Thorlund K, Ioannidis JP. Demystifying trial networks and network meta-analysis. Br Med J. 2013;346. 10.1136/bmj.f2914. 10.1136/bmj.f2914 23674332

[13] Harrer M, Cuijpers P, A FT, Ebert DD. Doing Meta-Analysis With R: A Hands-On Guide. 1st ed. Boca Raton and London: Chapman & Hall/CRC Press; 2021.

[14] Berlin JA, Santanna J, Schmid CH, Szczech LA, Feldman HI. Individual patient-versus group-level data meta-regressions for the investigation of treatment effect modifiers: ecological bias rears its ugly head. Stat Med. 2002;21(3):371–87. 11813224 10.1002/sim.1023

[15] Thompson SG, Higgins JP. How should meta-regression analyses be undertaken and interpreted? Stat Med. 2002;21(11):1559–73. 12111920 10.1002/sim.1187

[16] da Costa BR, Sutton AJ. A comparison of the statistical performance of different meta-analysis models for the synthesis of subgroup effects from randomized clinical trials. BMC Med Res Methodol. 2019;19:1–19. 31655550 10.1186/s12874-019-0831-8 PMC6815379

[17] Stewart LA, Tierney JF. To IPD or not to IPD? Advantages and disadvantages of systematic reviews using individual patient data. Eval Health Prof. 2002;25(1):76–97. 11868447 10.1177/0163278702025001006

[18] Riley RD, Dias S, Donegan S, Tierney JF, Stewart LA, Efthimiou O, et al. Using individual participant data to improve network meta-analysis projects. BMJ Evid-Based Med. 2023;28(3):197–203. 35948411 10.1136/bmjebm-2022-111931 PMC10313959

[19] Riley RD, Lambert PC, Abo-Zaid G. Meta-analysis of individual participant data: rationale, conduct, and reporting. British Med J. 2010;340:1–7. 10.1136/bmj.c221 20139215

[20] Burke DL, Ensor J, Riley RD. Meta-analysis using individual participant data: one-stage and two-stage approaches, and why they may differ. Stat Med. 2017;36(5):855–75. 27747915 10.1002/sim.7141 PMC5297998

[21] Kontopantelis E. A comparison of one-stage vs two-stage individual patient data meta-analysis methods: A simulation study. Res Synth Methods. 2018;9(3):417–30. 29786975 10.1002/jrsm.1303 PMC6175226

[22] Hua H, Burke DL, Crowther MJ, Ensor J, Tudur Smith C, Riley RD. One-stage individual participant data meta-analysis models: estimation of treatment-covariate interactions must avoid ecological bias by separating out within-trial and across-trial information. Stat Med. 2017;36(5):772–89. 27910122 10.1002/sim.7171 PMC5299543

[23] Riley RD, Simmonds MC, Look MP. Evidence synthesis combining individual patient data and aggregate data: a systematic review identified current practice and possible methods. J Clin Epidemiol. 2007;60(5):431–e1. 17419953 10.1016/j.jclinepi.2006.09.009

[24] Fisher DJ, Carpenter JR, Morris TP, Freeman SC, Tierney JF. Meta-analytical methods to identify who benefits most from treatments: daft, deluded, or deft approach? Br Med J. 2017;356:1–6. 10.1136/bmj.j573 PMC5421441 28258124

[25] Chaimani A, Caldwell DM, Li T, Higgins JPT, Salanti G. Chapter 11: Undertaking network meta-analyses [last updated October 2019]. In: Higgins JPT, Thomas J, Chandler J, Cumpston M, Li T, Page MJ, Welch VA (editors). Cochrane Handbook for Systematic Reviews of Interventions version 6.5. Cochrane. 2024. Available from https://www.training.cochrane.org/handbook.

[26] Wheaton L, Jackson D, Bujkiewicz S. Bayesian meta-analysis for evaluating treatment effectiveness in biomarker subgroups using trials of mixed patient populations. Res Synth Methods. 2024;15(4):543–60. 10.1002/jrsm.1707 38316618

[27] Sørensen AL, Marschner IC. Linear mixed models for investigating effect modification in subgroup meta-analysis. Stat Methods Med Res. 2023;32(5):994–1009. 36924263 10.1177/09622802231163330

[28] Godolphin PJ, White IR, Tierney JF, Fisher DJ. Estimating interactions and subgroup-specific treatment effects in meta-analysis without aggregation bias: A within-trial framework. Res Synth Methods. 2023;14(1):68–78. 35833636 10.1002/jrsm.1590 PMC10087172

[29] Godolphin P, White I, Tierney J, Fisher D. metafloat: A Stata package to estimate covariate interactions and subgroup-specific treatment effects in meta-analysis. 2023. Available on GitHub. https://github.com/UCL/metafloat. Accessed 13 Nov 2024.

[30] Efthimiou O, Mavridis D, Debray TP, Samara M, Belger M, Siontis GC, et al. Combining randomized and non-randomized evidence in network meta-analysis. Stat Med. 2017;36(8):1210–26. 28083901 10.1002/sim.7223

[31] Proctor T, Zimmermann S, Seide S, Kieser M. A comparison of methods for enriching network meta-analyses in the absence of individual patient data. Res Synth Methods. 2022;13(6):745–59. 35521904 10.1002/jrsm.1568

[32] Dias S, Ades AE, Welton NJ, Jansen JP, Sutton AJ. Network meta-analysis for decision-making. Wiley; 2018.

[33] Harari O, Soltanifar M, Cappelleri JC, Verhoek A, Ouwens M, Daly C, et al. Network meta-interpolation: Effect modification adjustment in network meta-analysis using subgroup analyses. Res Synth Methods. 2023;14(2):211–33. 36283960 10.1002/jrsm.1608

[34] Proctor T, Jensen K, Kieser M. Integrated evaluation of targeted and non-targeted therapies in a network meta-analysis. Biom J. 2020;62(3):777–89. 31544262 10.1002/bimj.201800322

[35] Umemneku-Chikere CM, Wheaton L, Poad H, Ray D, Andrade IC, Khan S, et al. Individual participant data from digital sources informed and improved precision in the evaluation of predictive biomarkers in Bayesian network meta-analysis. J Clin Epidemiol. 2023;164:96–103. 37918640 10.1016/j.jclinepi.2023.10.018

[36] Stan Development Team. RStan: the R interface to Stan. 2024. R package version 2.32.6. https://mc-stan.org/. Accessed 13 Nov 2024.

[37] Gabry J, Veen D. shinystan: Interactive Visual and Numerical Diagnostics and Posterior Analysis for Bayesian Models. 2022. R package version 2.6.0. https://github.com/stan-dev/shinystan. Accessed 13 Nov 2024.

[38] Neumann J, Zeindl-Eberhart E, Kirchner T, Jung A. Frequency and type of KRAS mutations in routine diagnostic analysis of metastatic colorectal cancer. Pathol-Res Pract. 2009;205(12):858–62. 19679400 10.1016/j.prp.2009.07.010

[39] Clark J, Glasziou P, Del Mar C, Bannach-Brown A, Stehlik P, Scott AM. A full systematic review was completed in 2 weeks using automation tools: a case study. J Clin Epidemiol. 2020;121:81–90. 32004673 10.1016/j.jclinepi.2020.01.008

[40] Vale CL, Fisher DJ, Godolphin PJ, Rydzewska LH, Boher JM, Burdett S, et al. Which patients with metastatic hormone-sensitive prostate cancer benefit from docetaxel: a systematic review and meta-analysis of individual participant data from randomised trials. Lancet Oncol. 2023;24(7):783–97. 37414011 10.1016/S1470-2045(23)00230-9 PMC7616350

[41] Umemneku-Chikere CM, Ayodele O, Soares M, Khan S, Abrams K, Owen R, et al. Comparative review of pharmacological therapies in individuals with HER2-positive advanced breast cancer with focus on hormone receptor subgroups. Front Oncol. 2022;12:943154. 36059633 10.3389/fonc.2022.943154 PMC9433866

[42] Evrenoglou T, Metelli S, Thomas JS, Siafis S, Turner RM, Leucht S, et al. Sharing information across patient subgroups to draw conclusions from sparse treatment networks. Biom J. 2024;66(3):2200316. 10.1002/bimj.202200316 PMC7617248 38637311

[43] Dias S, Sutton AJ, Welton NJ, Ades A. NICE DSU technical support document 3: heterogeneity: subgroups, meta-regression, bias and bias-adjustment. 2011; last updated April 2012; available from https://www.nicedsu.org.uk. 27905717

[44] Riley RD, Lambert PC, Staessen JA, Wang J, Gueyffier F, Thijs L, et al. Meta-analysis of continuous outcomes combining individual patient data and aggregate data. Stat Med. 2008;27(11):1870–93. 18069721 10.1002/sim.3165

